# Glycoengineering of pertuzumab and its impact on the pharmacokinetic/pharmacodynamic properties

**DOI:** 10.1038/srep46347

**Published:** 2017-04-11

**Authors:** Cheng Luo, Song Chen, Na Xu, Chi Wang, Wen bo Sai, Wei Zhao, Ying chun Li, Xiao jing Hu, Hong Tian, Xiang dong Gao, Wen bing Yao

**Affiliations:** 1 Jiangsu Key Laboratory of Druggability of Biopharmaceuticals, School of Life Science and Technology, China Pharmaceutical University, Nanjing, 210009 China; 2 Jiangsu Chia Tai Tianqing Pharmaceutical Co, Ltd, Nanjing, 210023 China

## Abstract

Pertuzumab is an antihuman HER2 antibody developed for HER2 positive breast cancer. Glycosylation profiles are always the important issue for antibody based therapy. Previous findings have suggested the impact of glycosylation profiles on the function of antibodies, like pharmacodynamics, antibody-dependent cellular cytotoxicity (ADCC) and complement-dependent cytotoxicity (CDC). However, the roles of fucose and sialic acid in the function of therapeutic antibodies still need further investigation, especially the role of sialic acid in nonfucosylated antibodies. This study focused on the pharmacokinetic and pharmacodynamic properties of pertuzumab after glycoengineering. Herein, nonfucosylated pertuzumab was produced in CHO^*FUT8*−/−^ cells, and desialylated pertuzumab was generated by enzymatic hydrolysis. Present data indicated that fucose was critical for ADCC activity by influencing the interaction between pertuzumab and FcγRIIIa, nevertheless removal of sialic acid increased the ADCC and CDC activity of pertuzumab. Meanwhile, regarding to sialic acid, sialidase hydrolysis directly resulted in asialoglycoprotein receptors (ASGPRs) dependent clearance in hepatic cells *in vitro*. The pharmacokinetic assay revealed that co-injection of asialofetuin can protect desialylated pertuzumab against ASGPRs-mediated clearance. Taken together, the present study elucidated the importance of fucose and sialic acid for pertuzumab, and also provided further understanding of the relationship of glycosylation/pharmacokinetics/pharmacodynamics of therapeutic antibody.

Breast cancer is the most commonly diagnosed malignancy in women worldwide. About 0.226 million new cases were diagnosed in 2012 and an estimated 0.23 million would be diagnosed among women in 2015^1^,^2^. Breast cancer is considered as the second most common cancer overall^1^,^3^. HER2, a member of the human epidermal growth factor receptor family, is over-expressed in approximately 15~30% breast cancer^4^,^5^,^6^,^7^. In particular, HER2 is implicated in the pathogenic mechanisms of certain types of breast cancer by promoting cell growth and survival^8^,^9^. Owing to their critical role in breast cancer, HER2 has become an important biomarker and target of anticancer therapy for breast cancer patients^5^,^10^,^11^. In 2009, Pertuzumab was developed by Genentech for the treatment of HER2-positive breast cancer^12^,^13^,^14^. Pertuzumab is a humanized HER2-specifical monoclonal antibody, which prevents the dimerization of HER2 with other HER receptors by binding to HER2 and subsequently leads to suppression of tumor growth^13^,^15^,^16^.

The development of monoclonal antibodies (mAbs) production technology enabled their application in clinic for the treatment of a wide range of diseases, especially cancer^17^,^18^,^19^. Typical antibodies are composed of a heavy and a light chain to form the constant region (Fc) and antigen binding region (Fab)^20^. Pharmacodynamic (PD) and pharmacokinetic (PK) properties of mAbs are influenced by various factors which may restrict their clinical applications. Typically, therapeutic mAbs are produced using mammalian cell lines, and because of post-translation glycosylation, the biological activities of mAbs quite correlate with glycosylation profiles^21^, precisely for the feature of the Fc domain. Fucosylation of the Fc region is implicated in the FcγRIIIa binding and affects ADCC activity^22^,^23^,^24^. The function of terminal sialylation or sialic acid capped galactose has been controversially discussed^22^,^25^,^26^,^27^,^28^,^29^,^30^. Galactose has been reported to influence FcγRIIIa binding while not affect ADCC activity. Although, many studies indicated the correlation between the levels of sialic acid and the CDC activities, but there is still have no agreement on their interactions with FcγR^29^,^31^,^32^. Until now, whether the correlations between mAbs and glycosylation profiles are positive or negative is not determined and is likely dependent on the properties of different antibodies^21^. However, antibody glycoengineering, such as the deletion of FUT8 gene, could enhance the FcγRIIIa binding affinity and lead to an increased ADCC activity^33^,^34^. On the other hand, the impact of glycosylation on the PK behavior of mAbs was also investigated among these years, especially for terminal sialic acid. It is found that the sialylation degree of a glycoprotein can play a significant role in the serum half-life^35^,^36^,^37^. Glycoproteins can be removed from the circulation via a sugar-based interaction with ASGPRs on hepatic cells^37^,^38^,^39^. Several studies have reported that the ASGPRs induced endocytosis can lead to the clearance of glycoproteins in circulation^36^,^40^,^41^. Sialic acid content varies depending on several factors including cell types and fermentation process, all these factors can result in different terminal sugar moieties of mAbs. Whereas, whether sialic acid could influence the PK behavior of mAbs is not clear. To this end, we have engineered pertuzumab by CRISPR-Case9 and enzymatic hydrolysis to better understand the roles of the fucose and sialic acid. Herein, this study indicated that fucose and sialic acid descended the ADCC activity of pertuzumab, while the removal of sialic acid could result in an increased CDC activity, but induced a relatively poorer PK behavior. Additionally, we found that sialic acid affected the PK/PD relationship of pertuzumab, and would be critical quality attributes (CQA) for mAbs production.

## Results

### Characterization of pertuzumab expressed in Glycoengineered CHO cells

**Figure 1:**
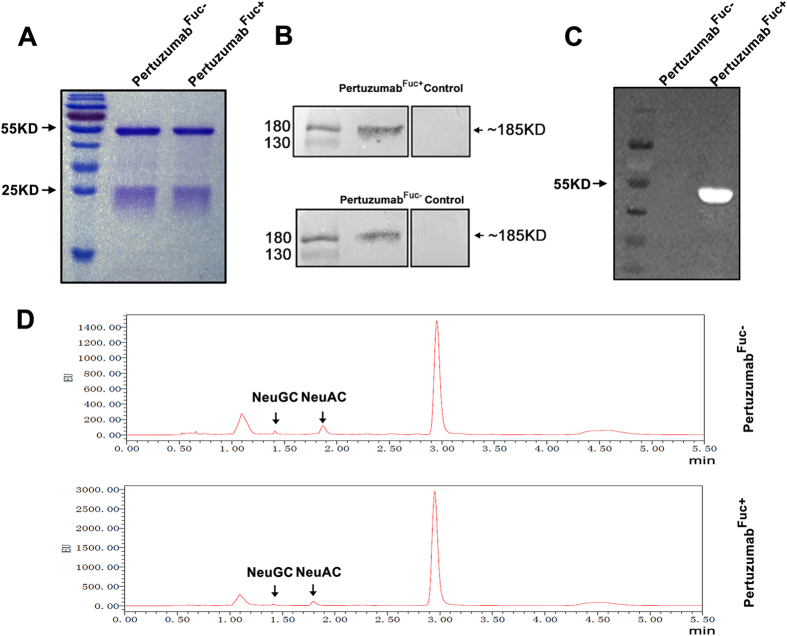
Biochemical analysis of glyco-modified pertuzumab from CHO-K1 and CHO^*FUT8*−/−^ cells. Antihuman HER2 antibodies were purified by Protein A chromatography, and analysis by (**A**) 10% SDS-PAGE, protein bands were stained by coomassie brilliant blue R250 reagent. (**B**) Western blot of the antihuman HER2 antibodies was detected by SK-BR-3 lysis using HRP-labeled goat antihuman Fc antibody and ECL. (**C**) Lectin blot was carried on PVDF membrane containing antihuman Her2 antibodies, and incubated with 5 μg/mL FITC-LCA for 2 h, then detected by Tanon Multi 5200. (**D**) RP-HPLC analysis of sialic acid. Fuc: fucose, SA: Sialic acid, NeuGC: N-acetylneuraminic, NeuAC: N-glycolylneuraminic acids.

To generate nonfucosylated pertuzumab, FUT8 gene was knocked out by CRISPR/Cas9 using LCA-selection method. Followed, the humanized pertuzumab glycovariants were produced from wild-type CHO-K1 cells and CHO^*FUT8*−/−^ cells using zeocin/blasticidin selection. Stably expressing cell pools were cultured for 5 days and the secreted antibodies were purified from the collected supernatants by Protein A affinity chromatography, and exchanged into PBS buffer using Desalting column. The heavy chain (~50 KD) and light chain (~25 KD) of concentrated antibodies were separated by SDS-PAGE (Fig. 1A). Western blot analysis showed that both pertuzumab^Fuc+^ and pertuzumab^Fuc−^ binds strongly with 185 KD HER2 protein from SK-BR-3 Cell lines (Fig. 1B). Then FITC-LCA lectin blot assay was performed to detect the fucose in the antibodies (Fig. 1C). As expected, pertuzumab derived from CHO^*FUT8*−/−^ cells displayed no visible fluorescence. For sialic acid determination, sialic acid was released and derivatized with DMB and analyzed by reversed-phase UPLC (Fig. 1D). The results showed the average sialic acid level for pertuzumab was 2.3~2.6% (mol/mol) (Table S1) and no significant difference were observed compared to nonfucosylated pertuzumab.

### *In vitro* binding properties of pertuzumab associated with the level of sialylation and fucosylation

**Figure 2:**
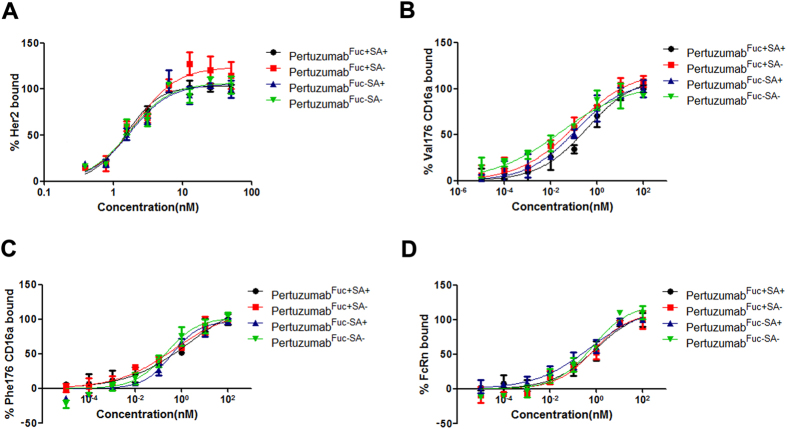
MST analysis of glyco-modified pertuzumab binding with HER2, CD16a and FcRn. Human HER2, CD16a, and FcRn were labeled by RED-NHS. Four glyco-modified pertuzumabs, pertuzumab^Fuc+SA+^ type (black dotted line), pertuzumab^Fuc+SA−^ type (red dotted line), pertuzumab^Fuc−SA+^ type (blue dotted line) and pertuzumab^Fuc−SA−^ type (green dotted line) were mixed with fluorophore-labeled ligands respectively. The thermophoretic mobility was measured by a MST machine. The binding data were analyzed by Prism. (**A**) HER2, (**B**) CD17a Val176, (**C**) CD16a Phe176 and (**D**) FcRn. Data are presented as mean values ± SD. Data are representative of three independent experiments with similar results (n = 3). Fuc: fucose, SA: Sialic acid.

Both the pertuzumab and nonfucosylated pertuzumab were desialylated by sialidase A to generate four glycoforms. Microscale thermophoresis (MST) method was used to determine the binding properties of the four pertuzumab glycoforms to HER2 and Fc receptors, respectively. For this purpose, HER2, CD16a and FcRn were fluorescently labeled. Accordingly, binding of pertuzumab to fluorophore-labeled molecules change the thermophoretic mobility, and resulted in a saturation binding curve for gradient pertuzumab concentration (Fig. 2A,B,C and D). All glycoforms of pertuzumab bound to HER2 and FcRn with a similar binding affinity (Fig. 2A and D, Table 1), whereas nonfucosylated pertuzumab showed significantly higher binding affinity to CD16a. Moreover, the removal of sialic acid can also significantly enhance the binding to CD16a (Fig. 2B and C, Table 1). All these demonstrated that the Fc fucose and sialic acid was critical for the binding to FcγRIIIa.

### Determination of ADCC activities of different glycoforms of pertuzumab

**Figure 3:**
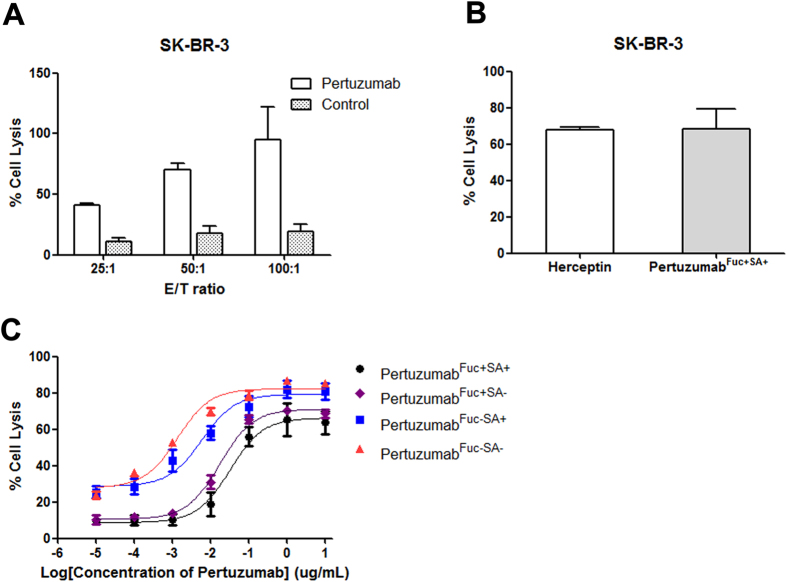
ADCC activity of glyco-modified pertuzumab. Cell lysis of SK-BR-3 was induced by human PBMCs in the presence of antihuman Her2 antibodies. (**A**) 10000 SK-BR-3 cells were incubated with human PBMCs at indicated E/T ratios with or without the presence of 10 μg/mL pertuzumab or (**B**) Herceptin for 4 h at 37 °C. LDH release was detected by absorbance at 490 nM for gradient glyco-pertuzumabs (**C**). The concentration of pertuzumab^Fuc+SA+^ type (black dotted line), pertuzumab^Fuc+SA−^ type (purple dotted line), pertuzumab^Fuc−SA+^ type (blue dotted line) and pertuzumab^Fuc−SA−^ type (red dotted line) were increased. The cell lysis was calculated according to the max target release well. Data are presented as mean values ± SD. Data are representative of three independent experiments with similar results (n = 3). Fuc: fucose, SA: Sialic acid.

HER2 positive cell line SK-BR-3 was used as target cells (T) to test the ADCC reactivity and peripheral blood mononuclear cells (PBMCs) from healthy human donors were chosen as effecter cells (E). First, different effecter and target ratios were tested (E:T) (Fig. 3A), and the increased cytotoxicity was clearly depend on the E:T ratio. 1 μg/mL Herceptin was also set as positive control and achieved equivalent ADCC activity with pertuzumab (Fig. 3B and Table S2). Further, four types of glyco-modified pertuzumabs were applied for the ADCC measurements (Fig. 3C). The result showed that 5~6 fold higher cytotoxicity was observed for pertuzumab^Fuc−SA+^ compared with pertuzumab^Fuc+SA+^, whereas pertuzumab^Fuc−SA−^ achieved a significant ~20 fold higher ADCC activity (Table S3). The incensement of ADCC reactivity for glyco-modified pertuzumabs correlated with the binding properties observed in MST assays. These results are in accordance with increased FcγRIIIa binding affinity on human NK cells for nonfucosylated and desialylated pertuzumab, and which resulted in higher ADCC activity.

### Correlation of CDC potency with different glycoforms of pertuzumab

**Figure 4:**
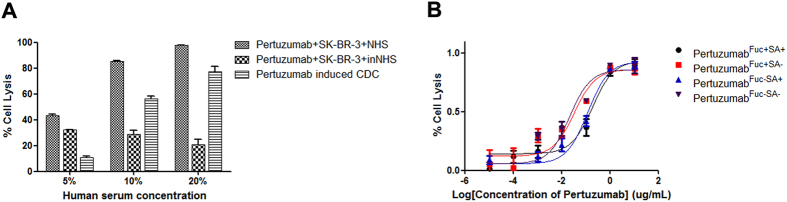
CDC activity of glyco-modified pertuzumab. Cell lysis of SK-BR-3 was induced by normal human serum in the presence of antihuman HER2 antibodies. (**A**) Human serum concentration was optimized for antihuman HER2 based CDC assay, (**B**) 20000 SK-BR-3 cells were pre-incubated with indicated concentration of four glyco-modified pertuzumabs at 37 °C for 30 min, 10% normal human serum was added and incubated for another 60 min. Cell viability was detected by ATP content. NHS, normal human serum; inNHS, inactived normal human serum. Data are presented as mean values ± SD. Data are representative of three independent experiments with similar results (n = 3). Fuc: fucose, SA: Sialic acid.

The CDC activities of four glyco-modified pertuzumabs against SK-BR-3 cell lines were measured using fresh human serum from healthy donors as a complement source (Fig. 4A). The cell viability decreased with raised concentration of human serum compared with inactivated human serum. Followed, 10% human serum was used to assess the CDC potency of pertuzumab. There was no significant difference between the fucose (+) type and the fucose (−) type of pertuzumab (Fig. 4B and Table S4). Meanwhile, an approximate 5 fold higher CDC activity was found for the sialic acid (−) type of pertuzumab, and pertuzumab^Fuc−SA−^ exhibited the highest CDC activity (Fig. 4B and Table S4).

### *In vitro* cellular clearance of Pertuzumab and its glycoform variants

**Figure 5:**
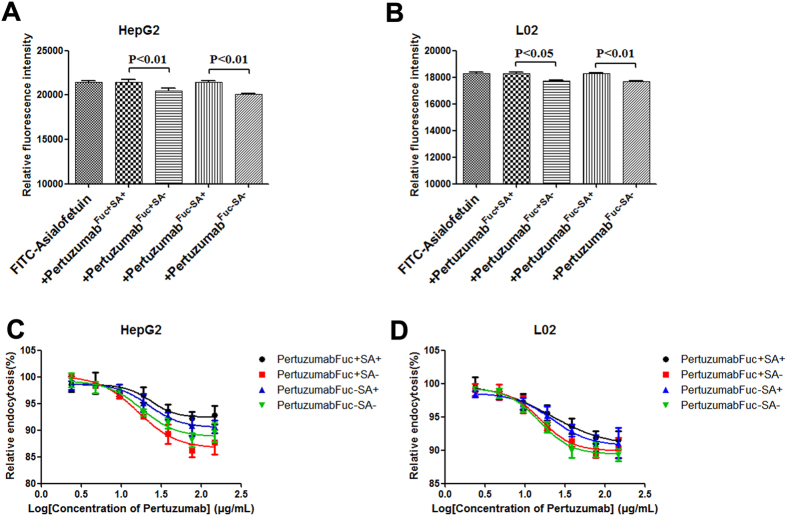
*In vitro* endocytosis of glyco-modified pertuzumab. Cells were incubated with 10 μM FITC-asialofetuin in the presence of 1 μM glyco-modified pertuzumabs. After 60 min incubation, endocytosis was stopped and intracellular FITC-asialofetuin was extracted to measure the fluorescence intensity (**A,B**). Indicated gradient glyco-modified pertuzumabs were added in the presence of 100 nM FITC-asialofetuin in HepG2 (**A**) and L-02 (**B**). Data are representative of three independent experiments with similar results (n = 3). Fuc: fucose, SA: Sialic acid.

Previous findings suggested that ASGPRs of hepatocytes could mediate the endocytosis of desialylated proteins and undergo intracellular degradation progress. We investigated the endocytosis properties of glyco-modified pertuzumabs. FITC labeled asialofetuin (ASF) was proved to be the ligand of ASGPRs^42^, and the endocytosis of pertuzumab was determined as the amount of intracellular FITC-ASF that internalized into hepatocytes. The data presented in Fig. 5A and B showed that the sialic acid (−) types of pertuzumab could significantly inhibit the endocytosis of FITC-ASF compared with sialic acid (+) types of pertuzumab, whereas the level of fucose was not seem to influence the endocytosis of FITC-ASF. Further, gradient concentrations of pertuzumab were used to antagonize the endocytosis of FITC-ASF (Fig. 5C and D). Although the percentage decline of FITC-ASF endocytosis was about 10%, desialylated pertuzumabs displayed more ability to suppress the endocytosis of FITC-ASF (Table S5), and these suggested that the sialic acid dependent ASGPRs interactions could play a role in the intracellular clearance of pertuzumab.

### Pharmacokinetic properties of the pertuzumab sialic acid variants in mice

**Figure 6:**
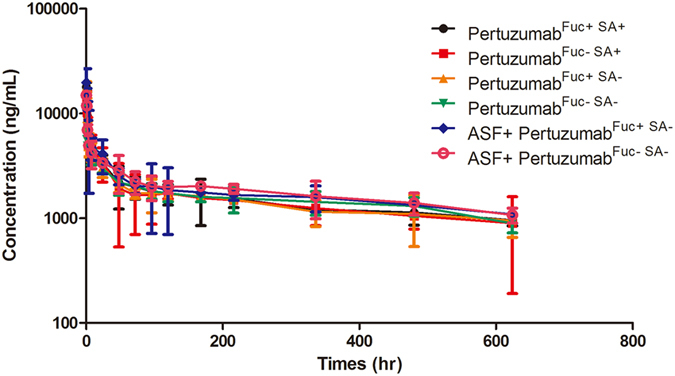
Plasma clearance of glyco-modified pertuzumab in mice. For the measurement of plasma clearance for glyco-modified pertuzumabs in mice, male ICR mice (n = 5) were injected intravenously with antihuman HER2 antibodies. The concentrations of antibodies in serum were measured by HER2 specific ELISA. The serum half-life was analyzed from the elimination phase. Pertuzumab^Fuc+SA+^ type (black dotted line), pertuzumab^Fuc−SA+^ type (red dotted line), pertuzumab^Fuc+SA−^ type (yellow dotted line), pertuzumab^Fuc−SA−^ type (green dotted line), pertuzumab^Fuc+SA−^ type + asialofetuin (blue dotted line) and pertuzumab^Fuc+SA−^ type + asialofetuin (pink dotted line). Data are representative of three independent experiments with similar results. Fuc: fucose, SA: Sialic acid.

Given the connection between fucose/sialic acid content and the *in vitro* cellular clearance properties of pertuzumab, we further characterized the impact of fucose and sialic acid content of pertuzumabs on their PK profiles. The PK of four types of pertuzumab was evaluated in ICR mice, and the free pertuzumab in mice serum were measured based on HER2 binding (Figure S2). 100 mg/kg Asialofetuin was intravenous injected 30 min before the experiment in sialic acid (−) groups (Fig. 6). The plasma concentration-time curve displayed a biphasic clearance profile — a rapid distribution phase and a longer elimination phase for each glyco-modified petuzumabs (Fig. 6). The distribution phase of four glyco-modified pertuzumabs was less than 3 h, the terminal elimination half-life was between 10 and 15 days. Compared with the sialic acid (+) type, the sialic acid (−) type pertuzumabs showed a significantly increased clearance values (~11% incensement, Table 2), which displayed an 11%~35% decreased half-life. Additionally, no significantly differences were observed for fucose (−) types of pertuzumab. To confirm whether the clearance differences of pertuzumabs was due to the interaction with ASGPRs *in vivo*, we pre-injected asialofetuin and evaluated the PK of sialic acid (−) types of pertuzumab. In asialofetuin pre-injected groups, the average clearance values decreased significantly, and the elimination half-life was prolonged (Table 2).

## Discussion

Many studies have shown that Fc glycosylation of monoclonal antibodies play a critical role for their biological activities^18^,^21^. However, few studies have addressed the effects of sialic acid on the PK/PD relationship of nonfucosylated antibodies. In this study, we employed the glycoengineering technique *in vitro* to generate four glyco-modified HER2 antibodies. In the present study, the anti-HER2 IgG1 amino acid sequence was equivalent to that of pertuzumab approved for the treatment of HER2 positive breast cancer (See supplemental sequences).

FUT8 is known to be the only gene to encode fucosyltransferase, which catalyzes the transfer of fucose from GDP-fucose to N-linked type complex glycoprotein. FUT8 knockout cell lines would be theoretically to produce nonfucosylated glycoprotein. To achieve this goal, CRISPR/Cas9 was employed previously to knockout the FUT8 gene in CHO-K1 cell using LCA-selection method, and lectin based blot was applied to detect the fucosylation of glycoprotein. We therefore chose the FUT8 knockout CHO cell line and wild-type CHO-K1 as host cell lines to generate pertuzumab respectively. Furthermore, we used sialidase A to produce sialic acid (−) type pertuzumab *in vitro* by enzymatic digestion for 48 h in 4 °C (Figure S1). Consequently, we succeeded to get four combinations on the fucose and sialic acid levels of pertuzumab. Binding affinities of four glyco-modified pertuzumabs were quantified and both nonfucosylated pertuzumab and desialylated pertuzumab showed the same binding affinity with HER2 antigen *in vitro*. Fucosylated pertuzumab showed a weaker binding to CD16a, and desialylated pertuzumab could enhance the CD16a binding. And these were consistent with previous reports^43^,^44^. In the ADCC assay, the nonfucosylated pertuzumab showed stronger ADCC reactivity. On the other hand, the strongest CD16a binding activity of pertuzumab^Fuc−SA−^ induced the strongest ADCC activity. Accordingly, the weakest binding activity of pertuzumab^Fuc+SA+^ resulted in a weakest ADCC activity. The differences of ADCC activity among these pertuzumab glycoforms suggested the negative role of fucose and sialic acid, for their roles in binding with FcγRIIIa *in vitro*, which subsequently influence the interaction with FcγRIIIa in NK cells *in vivo*. Followed, the CDC assay mediated by human serum was carried out. Desialylated pertuzumab induced a relatively higher CDC activity, whereas the fucose did not seem to affect the CDC activity for pertuzumab, and we believe the non-reducing end of N-linked oligosaccharides would be critical for C1q interaction rather than core-fucose.

Glycosylation impact on the PK properties of antibodies has not been well characterized in the previous literatures. Glycoproteins with exposed terminal sugar residues such as galactose are known to be internalized by ASGPRs on hepatocytes, which can be rapidly removed from circulation and led to a short half-life. So oligosaccharides of glycoprotein are considered to play a critical role in *in vivo* clearance of glycoprotein. In the case of glyco-antibodies, although human IgG1 antibodies can undergo an FcRn-mediated transcytosis, glycan-receptor mediated clearance did exist, including ASGPRs mediated^37^,^45^,^46^ and Mannose receptors mediated^47^,^48^,^49^ clearance. Desialylation are common during antibody production, and we demonstrated desialylated pertuzumab was more effectively internalized into hepatocytes *in vitro* using an endocytosis competition assay. Actually, the desialylated type of pertuzumab showed a more rapid clearance in mice, and co-injection of asialofetuin as an antagonistic agent for ASGPRs decreased the clearance rate significantly. And we confirmed that the desialylated pertuzumab from CHO^*FUT8*−/−^ had the shortest *in vivo* half-life in mice, and then desialylated pertuzumab from CHO-K1, nonfucosylated type and normal pertuzumab from CHO-K1, the clearance seemed to depend on the sialic acid levels. Meanwhile, a similar FcRn-binding affinity was observed among the four different glyco-modified pertuzumabs in our MST assay. This finding is consistent with previous reports that glycosylation may not influence the interaction with FcRn^50^,^51^,^52^. And they all displayed a relative long serum half-life in mice, about 10 days. Therefore, our findings are also reflecting the clearance profiles of human IgGs in human *in vivo*.

**Figure 7:**
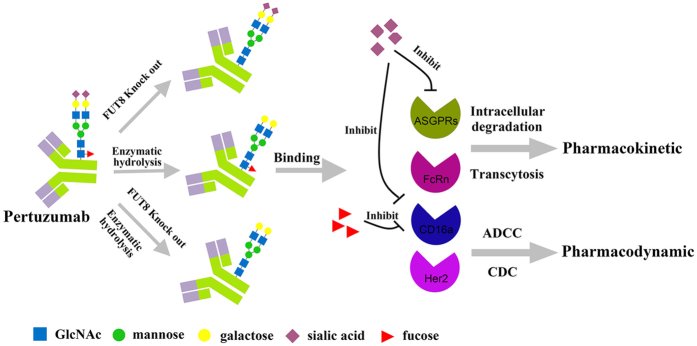
The impact of fucose and sialic acid on the pharmacokinetic and pharmacodynamic properties of pertuzumab.

In conclusion, as shown in Fig. 7, we used glycoengineering technique to generate four types of pertuzumabs by altering the fucosylation and sialylation. We found core fucose was important for the ADCC activity, and removal of terminal sialic acid could enhance both ADCC (2~4 fold) and CDC (~5 fold) activity of the pertuzumab, whereas the poorly sialylated pertuzumab also led a relatively higher clearance rate in mice and the co-injection of asialofetuin could protect the desialylated pertuzumab against ASGPRs induced endocytosis in hepatocytes.

## Materials and Methods

### Cell lines and cell culture

CHO^*FUT8*−/−^ cells were generated by CRISPR/Cas9 using LCA-selection method previously. The CHO cell lines containing antihuman HER2 antibody gene was cultured in DMEM/F12 (Gibco, China) selective medium supplemented with 10% fetal bovine serum (Gibco, USA), 1 μg/mL Zeocin (InvivoGen, USA) and 20 μg/mL Blasticidin (InvivoGen), and incubated at 37 °C, 5% CO_2_. Stably expressing cell pools were cultured in CD-CHO medium (Gibco, USA) in 1 L shake flask for 5 days to collect supernatants. In ADCC and CDC assays, SK-BR-3 was cultured in DMEM (Gibco) medium supplemented with 10% FBS.

### Preparation of glycoengineered pertuzumab

Two antihuman HER2 (pertuzumab) IgG1-producing clones from CHO^*FUT8*−/−^ or CHO-K1 were expanded into 1 L shake flask and cultured in serum-free medium CD-CHO at 37 °C, 5% CO_2_ for 5 days. Human IgG1 was purified from the cultured medium using Protein A column (GE, USA). The purified antibodies were exchanged into PBS (pH = 7.2) by a desalting column (GE), and concentrated using an Amicon Ultra-15 centrifugal filter (Millipore, USA). The obtained mAb was aliquoted and stored at −80 °C. Desialylated pertuzumab were generated by the removal of sialic acids using Arthrobacter ureafaciens Glyko Sialidase A (PROzyme, CA). Further, reversed-phase UPLC (detailed protocol see supplemental file) was used for the detection of sialic acid content in pertuzumab after hydrolyzed by acid and derived by DMB.

### Western blot and Lectin blot

Purified pertuzumabs were analyzed by 10% SDS-PAGE and transferred to a PVDF membrane. For Lectin blot, the membrane was blocked and incubated with 5 μg/mL FITC-LCA for 2 h at room temperature in dark. The fluorescence was detected by Tanon 5200 Multi (Tanon, China). Further, SK-BR-3 cells were lysed, the concentration of cell lysate was determined by Bradford method. 40 μg/well proteins were separated by 10% SDS-PAGE in non-reduced status, and transferred to a PVDF membrane. The membrane was blocked and incubated with purified anti-HER2 mAbs overnight at 4 °C. HRP-Mouse anti-Human IgG1 (Life Technologies, USA) was used to incubate for 1 h at room temperature following four washes with TBST, and the specific bands were detected using ECL detection system (Miliproe).

### MST assays

MicroScale Thermophoresis (MST, NanoTemper, Germany) was used to detect the affinity of pertuzumab with Human HER2, CD16a and FcRn. Before the MST assays, the ligands (HER2, Sino Biological, China; CD16a, R&D system, USA; FcRn, R&D system) were labeled according to the manufacturer’s instructions (Nano Temper) using Monolith NT protein labeling kit RED-NHS respectively. 10 μL labeled ligands were mixed with 10 μL gradient mAbs, and filled into capillaries to measure the fluorescence intensity in a Monolith NT.115 system. In the measuring, 40% was chosen for IR-laser power. The binding data were fit using the Hill equation in Prism (Graphpad, USA).

### Antibody-dependent cellular cytotoxicity (ADCC) assays

The collection of PBMCs and serum from healthy volunteers has been carried out in accordance with the Declaration of Helsinki, and was approved by the Institutional Review Board of Zhongda Hospital (Southeast University, Nanjing, China). A signed informed consent was obtained from each volunteer. ADCC activity was determined by the LDH release assay. SK-BR-3 was chosen as target cells (T). Human PBMCs purified from healthy donor using Lympholyte-H (Cedarlane, USA) were used as effector cells (E). E/T ratio was determined by mix SK-BR-3 with PBMCs in the presence of antihuman HER2 antibodies. Further, PBMCs mixed with SK-BR-3 at an E/T ration of 50/1, and incubated with different pertuzumab concentrations. After 4 h incubation at 37 °C., the plate was centrifuged at 250 g for 5 min and transferred 50 μL of the supernatants to another new 96-well plate. According the manufacturer’s instructions, 50 μL/well substrate was added to each well and incubate for 30 min, the reaction was stopped and read the absorbance of 490 nm. Besides, effector cells self-release, target cells self-release, target cells maximum release, blank control group and positive control group were also set.

### Complement-dependent cytotoxicity (CDC) assays

CDC activity was determined by ATP assay. SK-BR-3 cells acted as target cells, normal human serum was selected as complement resource. Firstly, different human serum dilutions (normal serum and inactive serum) were applied to incubate with SK-BR-3 in the presence of antihuman HER2 antibody. Followed, 10% normal human serum was chosen to investigate the CDC activity of different concentrations of pertuzumab. After 1 h incubation at 37 °C., CellTiter-Glo 2.0 reagent (Promega, USA) was used to measure the intracellular ATP content according to the manufacturer’s instructions.

### Endocytosis assays

Endocytosis assays were carried out using FITC-asialofetuin in HepG2 and L-02 cells. 1 × 10^4^ cells per well (96 well plate, Corning, USA) were seeded 24 h before, FITC-asialofetuin was incubated with HepG2 and L-02 in the presence of four glyco-modified pertuzumabs at 37 °C for 1 h. Then medium was discarded and the endocytosis was stopped by cold PBS. Cells were lysed to extract intracellular fluorescence, and the fluorescence intensity was measured by SAFIRE2 (Tecan, Switzerland).

### *In vitro* pharmacokinetics of pertuzumab

All experimental procedures with animals used in the present study were according to the Guide for the Care and Use of Laboratory Animals as adopted and promulgated by the United States National Institutes of Health, and had been given prior approval by the China Pharmaceutical Experimental Animal Manage Committee under Contract 2016 (su) −0020. For each glyco-modified pertuzumab, five 6-week-old male ICR mice (n = 5, Comparative Medicine Centre of Yangzhou University, China) were injected into tail vein (intravenous bolus) with 1 mg/kg antibody. For asialofetuin co-injection groups, 100 mg/kg asialofetuin was pre-injected 30 min before. Mice blood was collected from tail vein at time points: 0.5, 1, 2, 4, 8 and 24 h; and 2, 3, 4, 5, 7, 9, 14, 20, and 26 days, and serums were separated by centrifugation at 4000 rpm for 30 min at room temperature. The collected serums were stored at −80 °C for further analysis. The antibody concentration was measured by HER2 antibody specific ELISA, which coated with 100 ng human HER2 antigen per well, 200 ng/mL, 100 ng/mL, 50 ng/mL, 25 ng/mL, 12.5 ng/mL, 6.25 ng/mL, 3.125 ng/mL, 1.5625 ng/mL and 0 ng/mL pertuzumab were added as standards. The HER2-bound pertuzumab was detected by horseradish peroxidase (HRP) labeled mouse anti-human IgG Fc secondary antibody (Thermo Fisher). The concentrations of pertuzumab in the collected mouse serum were calculated according to the standard curve. The data were fit to a two-comparmental model, C(T) = A*EXP (−ALPHA*T) +B*EXP (−BETA*T), and PK parameters of AUC, CL, half-life, V1 and Vss were determined (Win Nonlin 5.2; Pharsight, USA).

### Statistics

Student’s t-test was used to calculate statistical significance between two groups. When more than two groups are compared One-Way- ANOVA analysis were applied. In all figures, error bars denote standard deviation.

## Additional Information

**How to cite this article**: Luo, C. *et al*. Glycoengineering of pertuzumab and its impact on the pharmacokinetic/pharmacodynamic properties. *Sci. Rep.* **7**, 46347; doi: 10.1038/srep46347 (2017).

**Publisher's note:** Springer Nature remains neutral with regard to jurisdictional claims in published maps and institutional affiliations.

## Supplementary Material

Supplementary Information

## Figures and Tables

**Table 1:** Binding affinity of antihuman HER2 antibodies to Human HER2, Phe176 CD16a, Val 176 CD16a and FcRn determined by MST.

	HER2		Phe176 CD16a		Val176 CD16a		FcRn	
	Kd(nM)	Fold*	Kd(nM)	Fold*	Kd(nM)	Fold*	Kd(nM)	Fold*
Pertuzumab^*Fuc*+*SA*+^	4.28 ± 0.21	1	1.31 ± 1.28	1	0.35 ± 0.25	1	0.86 ± 0.51	1
Pertuzumab^*Fuc*+*SA*−^	5.38 ± 0.62	0.79	0.45 ± 0.45	2.91	0.11 ± 0.06	3.18	0.83 ± 0.69	1.04
Pertuzumab^*Fuc*−*SA*+^	4.16 ± 0.36	1.02	0.29 ± 0.14	4.52	0.11 ± 0.06	3.18	0.84 ± 1.08	1.02
Pertuzumab^*Fuc*−*SA*−^	5.34 ± 0.45	0.80	0.13 ± 0.08	10.07	0.02 ± 0.02	17.50	0.82 ± 0.81	1.05

Data are presented as mean values ± SD. Data are representative of three independent experiments with similar results. Fuc: fucose, SA: Sialic acid.

*The “Fold” was calculated according to the value of Pertuzumab^*Fuc*+*SA*+^.

**Table 2:** Pharmacokinetic parameters of four glyco-modified pertuzumabs in ICR mice (n = 5) after a single 1 mg/kg intravenous administration.

	Species	Route	Doses (mg/kg)	AUC (hr*μg/mL)	CL (mL/day/kg)	V1 (mL/kg)	Vss (mL/kg)	Alpha_HL (day)	Beta_HL (day)
Pertuzumab^Fuc+SA+^	ICR	IV bolus	1	1327 ± 314.6	18.1 ± 4.3	57.3 ± 5.7	372.1 ± 50.4	0.08 ± 0.016	14.7 ± 4.8
Pertuzumab^Fuc−SA+^	ICR	IV bolus	1	1305.3 ± 321	18.4 ± 4.5	69.8 ± 7.1	406.1 ± 54.1	0.09 ± 0.019	15.7 ± 5.2
Pertuzumab^Fuc+SA−^	ICR	IV bolus	1	1202.2 ± 355.6	19.9 ± 5.9	55.1 ± 9.8	367.4 ± 62.3	0.05 ± 0.016	13.1 ± 5.2
Pertuzumab^Fuc−SA−^	ICR	IV bolus	1	1155.1 ± 251.4	20.7 ± 4.5	20.8 ± 9.4	313.5 ± 37.4	0.01 ± 0.0032	10.6 ± 2.9
ASF + Pertuzumab^Fuc+SA−^	ICR	IV bolus	100, 1	1569.1 ± 335.5	15.3 ± 3.3	44.7 ± 3.7	338.8 ± 38.6	0.07 ± 0.01	15.8 ± 4.5
ASF + Pertuzumab^Fuc−SA−^	ICR	IV bolus	100, 1	1624.5 ± 267.8	14.7 ± 2.4	54.1 ± 5.1	325.9 ± 27.1	0.05 ± 0.008	15.5 ± 3.3

Fuc: fucose, SA: Sialic acid, ASF: Asialofetuin, AUC: Area under the concentration–time curve from time zero extrapolated to infinity, CL: clearance, HL: half-life, *V*1: volume of distribution of the central compartment, *V*_ss_: steady state volume of distribution, IV: Intravenous. Data are presented as mean values ± SD.

Data are representative of three independent experiments with similar results.

## References

[b1] Siegel R. L., Miller K. D. & Jemal A. Cancer statistics, 2015. CA Cancer J Clin 65, 5–29, doi: 10.3322/caac.21254 (2015). 25559415

[b2] Ryerson A. B. . Annual Report to the Nation on the Status of Cancer, 1975–2012, featuring the increasing incidence of liver cancer. Cancer 122, 1312–1337, doi: 10.1002/cncr.29936 (2016). 26959385 PMC4840031

[b3] Incorvati J. A., Shah S., Mu Y. & Lu J. Targeted therapy for HER2 positive breast cancer. J Hematol Oncol 6, 38, doi: 10.1186/1756-8722-6-38 (2013). 23731980 PMC3703272

[b4] Cheang M. C. . Defining breast cancer intrinsic subtypes by quantitative receptor expression. Oncologist 20, 474–482, doi: 10.1634/theoncologist.2014-0372 (2015). 25908555 PMC4425383

[b5] Mitri Z., Constantine T. & O’Regan R. The HER2 Receptor in Breast Cancer: Pathophysiology, Clinical Use, and New Advances in Therapy. Chemother Res Pract 2012, 743193, doi: 10.1155/2012/743193 (2012). 23320171 PMC3539433

[b6] Burstein H. J. The distinctive nature of HER2-positive breast cancers. N Engl J Med 353, 1652–1654, doi: 10.1056/NEJMp058197 (2005). 16236735

[b7] Barnard M. E., Boeke C. E. & Tamimi R. M. Established breast cancer risk factors and risk of intrinsic tumor subtypes. Biochim Biophys Acta 1856, 73–85, doi: 10.1016/j.bbcan.2015.06.002 (2015). 26071880

[b8] Graus-Porta D., Beerli R. R., Daly J. M. & Hynes N. E. ErbB-2, the preferred heterodimerization partner of all ErbB receptors, is a mediator of lateral signaling. EMBO J 16, 1647–1655, doi: 10.1093/emboj/16.7.1647 (1997). 9130710 PMC1169769

[b9] Lohrisch C. & Piccart M. HER2/neu as a predictive factor in breast cancer. Clin Breast Cancer 2, 129–135; discussion 136–127, doi: 10.3816/CBC.2001.n.017 (2001). 11899784

[b10] Kos Z. & Dabbs D. J. Biomarker assessment and molecular testing for prognostication in breast cancer. Histopathology 68, 70–85, doi: 10.1111/his.12795 (2016). 26768030

[b11] Ross J. S. Breast cancer biomarkers and HER2 testing after 10 years of anti-HER2 therapy. Drug News Perspect 22, 93–106, doi: 10.1358/dnp.2009.22.2.1334452 (2009). 19330168

[b12] Swain S. M. . Pertuzumab, trastuzumab, and docetaxel for HER2-positive metastatic breast cancer (CLEOPATRA study): overall survival results from a randomised, double-blind, placebo-controlled, phase 3 study. Lancet Oncol 14, 461–471, doi: 10.1016/S1470-2045(13)70130-X (2013). 23602601 PMC4076842

[b13] de Bono J. S. . Open-label phase II study evaluating the efficacy and safety of two doses of pertuzumab in castrate chemotherapy-naive patients with hormone-refractory prostate cancer. J Clin Oncol 25, 257–262, doi: 10.1200/JCO.2006.07.0888 (2007). 17235043

[b14] Leung K. 111In-Diethylenetriamine pentaacetic acid-pertuzumab. Molecular Imaging and Constrast Agent Database (MICAD) (2004). 20641430

[b15] Nahta R., Hung M. C. & Esteva F. J. The HER-2-targeting antibodies trastuzumab and pertuzumab synergistically inhibit the survival of breast cancer cells. Cancer Res 64, 2343–2346 (2004). 15059883 10.1158/0008-5472.can-03-3856

[b16] Sendur M. A., Aksoy S. & Altundag K. Pertuzumab in HER2-positive breast cancer. Curr Med Res Opin 28, 1709–1716, doi: 10.1185/03007995.2012.728132 (2012). 22953713

[b17] Aires da Silva F., Corte-Real S. & Goncalves J. Recombinant antibodies as therapeutic agents: pathways for modeling new biodrugs. BioDrugs 22, 301–314 (2008). 18778112 10.2165/00063030-200822050-00003

[b18] Jefferis R. Glycosylation as a strategy to improve antibody-based therapeutics. Nat Rev Drug Discov 8, 226–234, doi: 10.1038/nrd2804 (2009). 19247305

[b19] Butler M. & Meneses-Acosta A. Recent advances in technology supporting biopharmaceutical production from mammalian cells. Appl Microbiol Biotechnol 96, 885–894, doi: 10.1007/s00253-012-4451-z (2012). 23053101 PMC7080107

[b20] Weiner L. M., Surana R. & Wang S. Monoclonal antibodies: versatile platforms for cancer immunotherapy. Nat Rev Immunol 10, 317–327, doi: 10.1038/nri2744 (2010). 20414205 PMC3508064

[b21] Liu L. Antibody glycosylation and its impact on the pharmacokinetics and pharmacodynamics of monoclonal antibodies and Fc-fusion proteins. J Pharm Sci 104, 1866–1884, doi: 10.1002/jps.24444 (2015). 25872915

[b22] Shinkawa T. . The absence of fucose but not the presence of galactose or bisecting N-acetylglucosamine of human IgG1 complex-type oligosaccharides shows the critical role of enhancing antibody-dependent cellular cytotoxicity. J Biol Chem 278, 3466–3473, doi: 10.1074/jbc.M210665200 (2003). 12427744

[b23] Malphettes L. . Highly efficient deletion of FUT8 in CHO cell lines using zinc-finger nucleases yields cells that produce completely nonfucosylated antibodies. Biotechnol Bioeng 106, 774–783, doi: 10.1002/bit.22751 (2010). 20564614

[b24] Isoda Y. . Importance of the Side Chain at Position 296 of Antibody Fc in Interactions with FcgammaRIIIa and Other Fcgamma Receptors. PLoS One 10, e0140120, doi: 10.1371/journal.pone.0140120 (2015). 26444434 PMC4596520

[b25] Boyd P. N., Lines A. C. & Patel A. K. The effect of the removal of sialic acid, galactose and total carbohydrate on the functional activity of Campath-1H. Mol Immunol 32, 1311–1318 (1995). 8643100 10.1016/0161-5890(95)00118-2

[b26] Hodoniczky J., Zheng Y. Z. & James D. C. Control of recombinant monoclonal antibody effector functions by Fc N-glycan remodeling *in vitro*. Biotechnol Prog 21, 1644–1652, doi: 10.1021/bp050228w (2005). 16321047

[b27] Houde D., Peng Y., Berkowitz S. A. & Engen J. R. Post-translational modifications differentially affect IgG1 conformation and receptor binding. Mol Cell Proteomics 9, 1716–1728, doi: 10.1074/mcp.M900540-MCP200 (2010). 20103567 PMC2938052

[b28] Kumpel B. M., Wang Y., Griffiths H. L., Hadley A. G. & Rook G. A. The biological activity of human monoclonal IgG anti-D is reduced by beta-galactosidase treatment. Hum Antibodies Hybridomas 6, 82–88 (1995). 8597627

[b29] Scallon B. J., Tam S. H., McCarthy S. G., Cai A. N. & Raju T. S. Higher levels of sialylated Fc glycans in immunoglobulin G molecules can adversely impact functionality. Mol Immunol 44, 1524–1534, doi: 10.1016/j.molimm.2006.09.005 (2007). 17045339

[b30] Stadlmann J., Pabst M. & Altmann F. Analytical and Functional Aspects of Antibody Sialylation. J Clin Immunol 30 Suppl 1, S15–19, doi: 10.1007/s10875-010-9409-2 (2010). 20390325 PMC2883086

[b31] Naso M. F., Tam S. H., Scallon B. J. & Raju T. S. Engineering host cell lines to reduce terminal sialylation of secreted antibodies. MAbs 2, 519–527, doi: 10.4161/mabs.2.5.13078 (2010). 20716959 PMC2958573

[b32] Kapur R., Einarsdottir H. K. & Vidarsson G. IgG-effector functions: “the good, the bad and the ugly”. Immunol Lett 160, 139–144, doi: 10.1016/j.imlet.2014.01.015 (2014). 24495619

[b33] Grav L. M. . One-step generation of triple knockout CHO cell lines using CRISPR/Cas9 and fluorescent enrichment. Biotechnol J 10, 1446–1456, doi: 10.1002/biot.201500027 (2015). 25864574

[b34] Ronda C. . Accelerating genome editing in CHO cells using CRISPR Cas9 and CRISPy, a web-based target finding tool. Biotechnol Bioeng 111, 1604–1616, doi: 10.1002/bit.25233 (2014). 24827782 PMC4312910

[b35] Liu L. . The impact of glycosylation on the pharmacokinetics of a TNFR2:Fc fusion protein expressed in Glycoengineered Pichia Pastoris. Pharm Res 30, 803–812, doi: 10.1007/s11095-012-0921-3 (2013). 23135825

[b36] Appa R. . Investigating clearance mechanisms for recombinant activated factor VII in a perfused liver model. Thromb Haemost 104, 243–251, doi: 10.1160/TH09-10-0723 (2010). 20508904

[b37] Ong G. L. . Galactose-conjugated antibodies in cancer therapy: properties and principles of action. Cancer Res 51, 1619–1626 (1991). 1998953

[b38] Hildenbrandt G. R. & Aronson N. N. Jr. Endocytosis of bovine lactoperoxidase by two carbohydrate-specific receptors in rat liver. Arch Biochem Biophys 237, 1–10 (1985). 2578766 10.1016/0003-9861(85)90247-4

[b39] Ashwell G. & Harford J. Carbohydrate-specific receptors of the liver. Annu Rev Biochem 51, 531–554, doi: 10.1146/annurev.bi.51.070182.002531 (1982). 6287920

[b40] Sodetz J. M., Pizzo S. V. & McKee P. A. Relationship of sialic acid to function and *in vivo* survival of human factor VIII/von Willebrand factor protein. J Biol Chem 252, 5538–5546 (1977). 301877

[b41] Seested T., Nielsen H. M., Christensen E. I. & Appa R. S. The unsialylated subpopulation of recombinant activated factor VII binds to the asialo-glycoprotein receptor (ASGPR) on primary rat hepatocytes. Thromb Haemost 104, 1166–1173, doi: 10.1160/TH10-06-0356 (2010). 20886190

[b42] Luo C. . Establishment of a fluorescence-based method to evaluate endocytosis of desialylated glycoproteins *in vitro*. Biomed Pharmacother 88, 87–94, doi: 10.1016/j.biopha.2016.12.085 (2017). 28095357

[b43] Kircheis R. . Correlation of ADCC activity with cytokine release induced by the stably expressed, glyco-engineered humanized Lewis Y-specific monoclonal antibody MB314. MAbs 4, 532–541, doi: 10.4161/mabs.20577 (2012). 22665069 PMC3499347

[b44] Thomann M. . *In vitro* glycoengineering of IgG1 and its effect on Fc receptor binding and ADCC activity. PLoS One 10, e0134949, doi: 10.1371/journal.pone.0134949 (2015). 26266936 PMC4534130

[b45] Sharma S. K. . Galactosylated antibodies and antibody-enzyme conjugates in antibody-directed enzyme prodrug therapy. Cancer 73, 1114–1120 (1994). 8306255 10.1002/1097-0142(19940201)73:3+<1114::aid-cncr2820731352>3.0.co;2-l

[b46] Mattes M. J. Biodistribution of antibodies after intraperitoneal or intravenous injection and effect of carbohydrate modifications. J Natl Cancer Inst 79, 855–863 (1987). 2443739

[b47] Kanda Y. . Comparison of biological activity among nonfucosylated therapeutic IgG1 antibodies with three different N-linked Fc oligosaccharides: the high-mannose, hybrid, and complex types. Glycobiology 17, 104–118, doi: 10.1093/glycob/cwl057 (2007). 17012310

[b48] Yu M. . Production, characterization, and pharmacokinetic properties of antibodies with N-linked mannose-5 glycans. MAbs 4, 475–487, doi: 10.4161/mabs.20737 (2012). 22699308 PMC3499342

[b49] Goetze A. M. . High-mannose glycans on the Fc region of therapeutic IgG antibodies increase serum clearance in humans. Glycobiology 21, 949–959, doi: 10.1093/glycob/cwr027 (2011). 21421994

[b50] Liu L. . Pharmacokinetics of IgG1 monoclonal antibodies produced in humanized Pichia pastoris with specific glycoforms: a comparative study with CHO produced materials. Biologicals 39, 205–210, doi: 10.1016/j.biologicals.2011.06.002 (2011). 21723741

[b51] Hobbs S. M., Jackson L. E. & Hoadley J. Interaction of aglycosyl immunoglobulins with the IgG Fc transport receptor from neonatal rat gut: comparison of deglycosylation by tunicamycin treatment and genetic engineering. Mol Immunol 29, 949–956 (1992). 1635563 10.1016/0161-5890(92)90133-i

[b52] Kim J. K., Tsen M. F., Ghetie V. & Ward E. S. Localization of the site of the murine IgG1 molecule that is involved in binding to the murine intestinal Fc receptor. Eur J Immunol 24, 2429–2434, doi: 10.1002/eji.1830241025 (1994). 7925571

