# Supplementary material for: Glycoengineering of pertuzumab and its impact on the pharmacokinetic/pharmacodynamic properties
Source: Sci Rep. 2017 Apr 11;7:46347. doi: 10.1038/srep46347 (PMC5387714; doi:10.1038/srep46347)
Supplement: Supplementary Information [file srep46347-s1.doc]

# Glycoengineering of pertuzumab and its impact on the pharmacokinetic/pharmacodynamic properties

Cheng L^uo^#a, Song Ch^en^#a, Na ^X^ua, Chi Wa^n^ga, Wen bo S^a^ia, Wei Zh^a^ob, Ying chun ^L^ib, Xiao jing ^H^ub, Hong Ti^a^nb, Xiang dong G^ao^a*, Wen bing Yao^a*^

^a^ *Jiangsu Key Laboratory of Druggability of Biopharmaceuticals, School of Life Science and Technology, China Pharmaceutical University, Nanjing, 210009 China*

^b^ *Jiangsu Chia Tai Tianqing Pharmaceutical Co, Ltd, Nanjing, 210023 China*

** Corresponding authors:*

*Xiangdong Gao,e-mail:xdgao@cpu.edu.cn, Telephone: +86-25-83271543, Fax: +86-25-83271249,*

Wenbing Yao*,e-mail:wbyao@cpu.edu.cn., Telephone: 86-25-83271218, Fax: +86-25-83302827*

# These authors contributed equally to this work

**Table S1**

Sialic acid level of antihuman Her2 antibodies determined by RP-HPLC

|  | NeuAC (Mol/Mol) | NeuGC (Mol/Mol) |
| --- | --- | --- |
| **Pertuzumab^Fuc+^** | 2.21 | 0.13 |
| **Pertuzumab^Fuc-^** | 2.46 | 0.13 |

Fuc: fucose

**Table S2**

Percentage of Pertuzumab and Herceptin induced human PBMCs mediated ADCC to SK-BR-3 cells

| E/T | Pertuzumab | Herceptin |
| --- | --- | --- |
| 25:1 | 29.9±2.85 | - |
| 50:1 | 53.03±1.45 | 51.01±6.46 |
| 100:1 | 75.22±30.77 | - |

Data are presented as mean values ± SD.

Data are representative of three independent experiments with similar results (n=3)

**Table S3**

Percentage of four glyco-modified pertuzumabs induced human PBMCs mediated ADCC to SK-BR-3 cells

|  | EC_50_(ng/mL) | Fold* |
| --- | --- | --- |
| Pertuzumab*^Fuc+SA+^* | 32.03 | 1 |
| Pertuzumab*^Fuc+SA-^* | 17.4 | 1.84 |
| Pertuzumab*^Fuc-SA+^* | 5.85 | 5.48 |
| Pertuzumab*^Fuc-SA-^* | 1.34 | 23.90 |

Data are representative of three independent experiments with similar results (n=3). Fuc: fucose, SA: sialic acid

*:The “Fold” was calculated according to the value of Pertuzumab*^Fuc+SA+^*.

**Table S4**

Percentage of four glyco-modified pertuzumabs induced human normal serum mediated CDC to SK-BR-3 cells

|  | EC_50_(ng/mL) | Fold* |
| --- | --- | --- |
| Pertuzumab*^Fuc+SA+^* | 18.39 | 1 |
| Pertuzumab*^Fuc+SA-^* | 3.32 | 5.54 |
| Pertuzumab*^Fuc-SA+^* | 11.68 | 1.57 |
| Pertuzumab*^Fuc-SA-^* | 2.12 | 8.67 |

Data are representative of three independent experiments with similar results (n=3). Fuc: fucose, SA: sialic acid

*:The “Fold” was calculated according to the value of Pertuzumab*^Fuc+SA+^*.

**Table S5**

Endocytosis of four glyco-modified pertuzumabs by HepG2 and L-02

|  | IC_50_(μg/mL) | |
| --- | --- | --- |
|  | HepG2 | L02 |
| Pertuzumab*^Fuc+SA+^* | 23.55±1.45 | 20.51±1.74 |
| Pertuzumab*^Fuc+SA-^* | 16.3±1.26 | 15.69±1.24 |
| Pertuzumab*^Fuc-SA+^* | 22.52±1.31 | 21.97±1.44 |
| Pertuzumab*^Fuc-SA-^* | 17.96±1.28 | 15.07±1.18 |

Data are presented as mean values ± SD. Fuc: fucose, SA: sialic acid

Data are representative of three independent experiments with similar results (n=3)

**The Gene code anti-HER2 IgG1 was cloned to pFUSEss plasmid by GenScript (GenScript, China), whose amino acid sequence was equivalent to that of Pertuzumab**

Light Chain:

GAATTCAGATATTCAGATGACCCAGAGCCCTTCTTCACTGTCCGCCAGCGTCGGAGATAGAGTCACAATCACCTGTAAAGCCAGCCAGGATGTCTCTATCGGAGTGGCATGGTATCAGCAGAAGCCTGGCAAAGCCCCTAAGCTGCTGATTTATTCTGCTAGTTACAGGTATACAGGCGTCCCCAGTCGGTTCTCAGGCTCCGGAAGCGGGACTGACTTTACCCTGACCATCTCCTCCCTCCAGCCAGAGGATTTCGCCACCTACTATTGCCAGCAGTACTATATCTACCCCTATACTTTTGGTCAGGGCACCAAAGTGGAAATTAAGCGTACGGTGGCTGCACCATCTGTCTTCATCTTCCCGCCATCTGATGAGCAGTTGAAATCTGGAACTGCCTCTGTTGTGTGCCTGCTGAATAACTTCTATCCCAGAGAGGCCAAAGTACAGTGGAAGGTGGATAACGCCCTCCAATCGGGTAACTCCCAGGAGAGTGTCACAGAGCAGGACAGCAAGGACAGCACCTACAGCCTCAGCAGCACCCTGACGCTGAGCAAAGCAGACTACGAGAAACACAAAGTCTACGCCTGCGAAGTCACCCATCAGGGCCTGAGCTCGCCCGTCACAAAGAGCTTCAACAGGGGAGAGTGTTAGAGGGAG

Heavy Chain:

GAATTCGGAGGTGCAGCTGGTGGAGAGTGGTGGTGGACTGGTGCAGCCCGGTGGGAGCCTGAGACTGAGTTGTGCCGCATCTGGATTTACTTTCACCGACTACACAATGGATTGGGTCAGACAGGCCCCTGGCAAGGGTCTGGAGTGGGTGGCCGATGTCAACCCTAATTCTGGCGGAAGTATCTACAACCAGAGGTTCAAGGGCCGGTTTACACTGTCAGTGGACAGGTCCAAAAACACTCTGTATCTCCAGATGAACTCCCTGAGAGCCGAAGATACCGCTGTCTACTATTGCGCTCGCAATCTGGGCCCCTCCTTCTACTTTGACTATTGGGGCCAGGGAACTCTGGTGACCGTCTCCAGCGCTAGCACCAAGGGCCCATCGGTCTTCCCCCTGGCACCCTCCTCCAAGAGCACCTCTGGGGGCACAGCGGCCCTGGGCTGCCTGGTCAAGGACTACTTCCCCGAACCGGTGACGGTGTCGTGGAACTCAGGCGCCCTGACCAGCGGCGTGCACACCTTCCCGGCTGTCCTACAGTCCTCAGGACTCTACTCCCTCAGCAGCGTGGTGACCGTGCCCTCCAGCAGCTTGGGCACCCAGACCTACATCTGCAACGTGAATCACAAGCCCAGCAACACCAAGGTGGACAAGAAAGTTGAGCCCAAATCTTGTGACAAAACTCACACATGCCCACCGTGCCCAGCACCTGAACTCCTGGGGGGACCGTCAGTCTTCCTCTTCCCCCCAAAACCCAAGGACACCCTCATGATCTCCCGGACCCCTGAGGTCACATGCGTGGTGGTGGACGTGAGCCACGAAGACCCTGAGGTCAAGTTCAACTGGTACGTGGACGGCGTGGAGGTGCATAATGCCAAGACAAAGCCGCGGGAGGAGCAGTACAACAGCACGTACCGTGTGGTCAGCGTCCTCACCGTCCTGCACCAGGACTGGCTGAATGGCAAGGAGTACAAGTGCAAGGTCTCCAACAAAGCCCTCCCAGCCCCCATCGAGAAAACCATCTCCAAAGCCAAAGGGCAGCCCCGAGAACCACAGGTGTACACCCTGCCCCCATCCCGGGAGGAGATGACCAAGAACCAGGTCAGCCTGACCTGCCTGGTCAAAGGCTTCTATCCCAGCGACATCGCCGTGGAGTGGGAGAGCAATGGGCAGCCGGAGAACAACTACAAGACCACGCCTCCCGTGCTGGACTCCGACGGCTCCTTCTTCCTCTACAGCAAGCTCACCGTGGACAAGAGCAGGTGGCAGCAGGGGAACGTCTTCTCATGCTCCGTGATGCATGAGGCTCTGCACAACCACTACACGCAGAAGAGCCTCTCCCTGTCTCCGGGTAAATGAGTCCTAGCTGG

**Figure S1 Time courses of desialylation of pertuzumab**


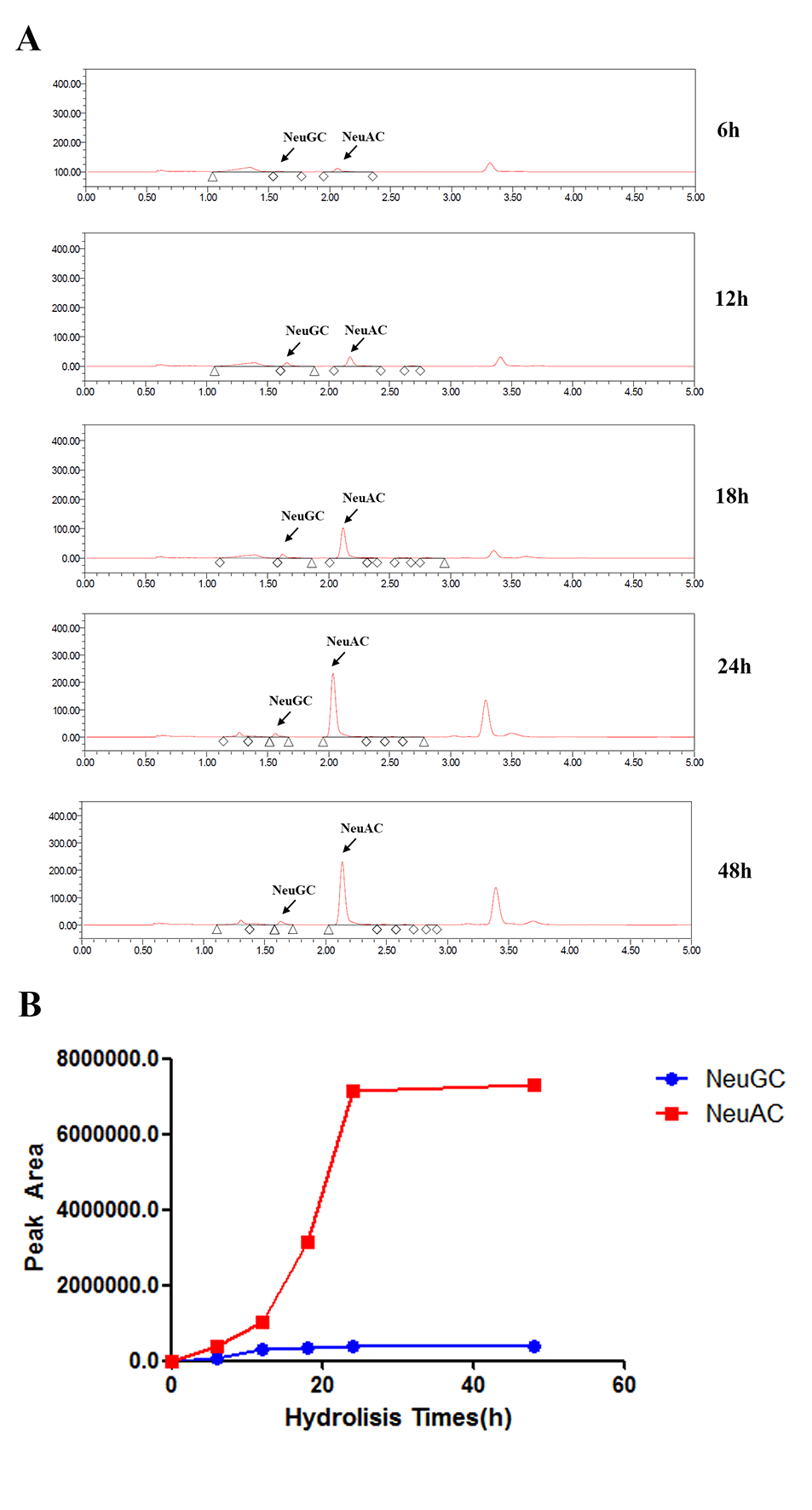


**Figure S2 Calibration Curve for Pertuzumab determination by Elisa assay**

**Supplemental Experimental Procedures**

**Sialic acid measurement by RP-UPLC**

20μg vacuum-dried antibodies were desialylated in the presence of 200μL 8 M aqueous acetic acid for 3 h at 80^o^C, 300rpm. The protein material was then cooled to room temperature, centrifuged at 10,000 g for 10 min at 4^o^C. The supernatant was then vacuum-dried. 60μL 1,2-Diamino-4,5-methylenedioxybenzene dihydrochloride (DMB) labeling solution (1mL contain 1.6 mg DMB, 3.2 mg sodium dithionite, 58 μL beta-mercaptoethanol, 82 μL acetic acid and 860 μL ultra-pure water) was added to each sample. The labeling reaction was conducted at 50 ^o^C for 2.5 h, 300 rpm, in dark. The reaction was stopped by adding 40μL ultra-pure water, and cooled to room temperature in the dark. The derivatives were separated by reversed-phase HPLC onto a 250 mm length × 4.6 mm C18 column (TOSOH ODS-120T, 250*4.6mm, 5μm, TOSOH, Japan),equilibrated at 40 ^o^C in acetonitrile:methanol:water (9%:7%:84%; v:v:v) . The elution products were monitored at λ_excitation_. = 373 nm/λ_emission_ = 448 nm using a fluorimetric detector (UPLC-FLR, Waters, USA). The elution was carried out at 0.9 mL/min, at 40 ^o^C for 1h.
